# Supplementary material for: A secreted protease-like protein in Zymoseptoria tritici is responsible for avirulence on Stb9 resistance gene in wheat
Source: PLoS Pathog. 2023 May 12;19(5):e1011376. doi: 10.1371/journal.ppat.1011376 (PMC10208482; doi:10.1371/journal.ppat.1011376)
Supplement: S1 Fig — (PDF) [file ppat.1011376.s008.pdf]

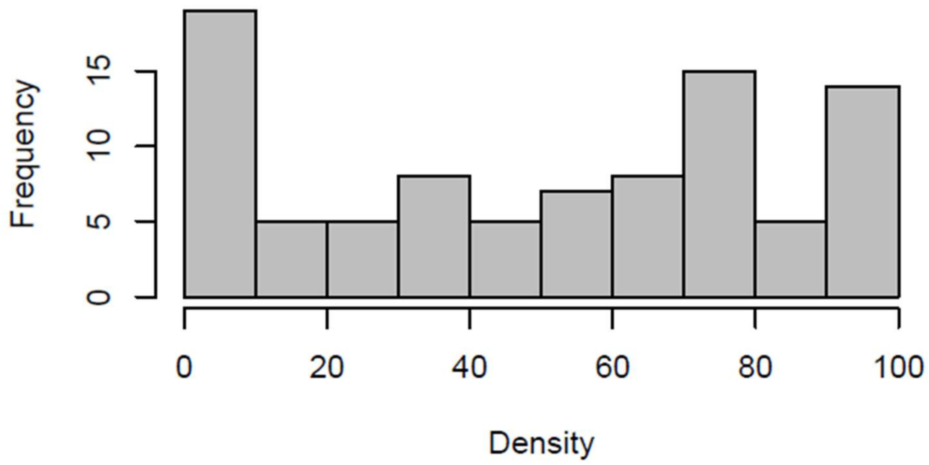

S1 Fig. Density distribution of percentage of leaf area covered by pycnidia (PLACP) measured at 21 days post inoculation (dpi) on the wheat cultivar 'Soissons' in the *Z. tritici* fungal population.
