# Supplementary material for: A secreted protease-like protein in Zymoseptoria tritici is responsible for avirulence on Stb9 resistance gene in wheat
Source: PLoS Pathog. 2023 May 12;19(5):e1011376. doi: 10.1371/journal.ppat.1011376 (PMC10208482; doi:10.1371/journal.ppat.1011376)
Supplement: S2 Fig — (a) Principal component analysis using full SNP data. PC1, PC2 and PC3 explained 1.30%, 1.12% an 0.69% of the phenotypic variation, respectively. (b) Heatmap of the kinship matrix using VanRaden method [55]. (PDF) [file ppat.1011376.s009.pdf]

(a)

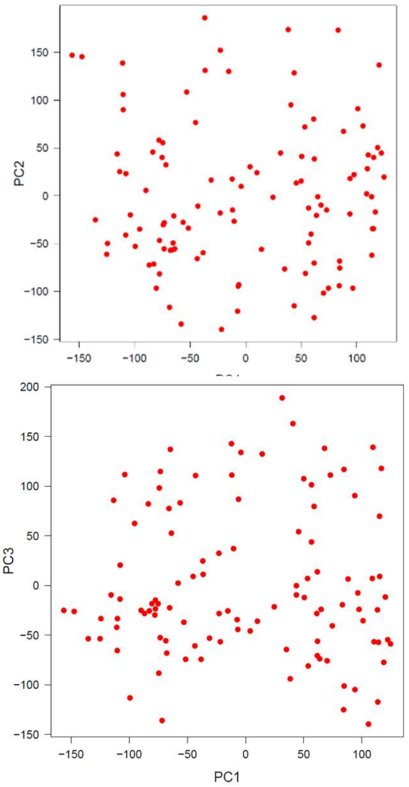

(b)

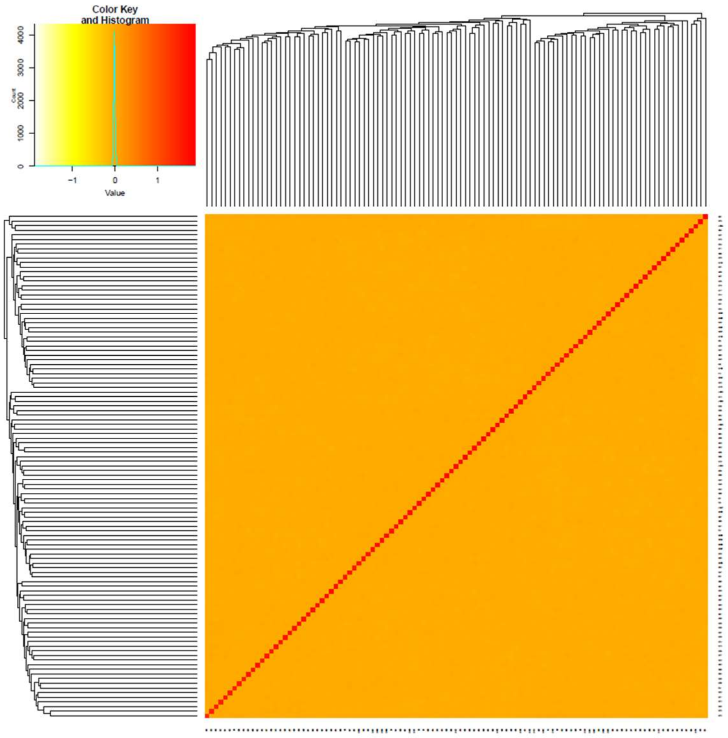

S2 Fig. Population structure and relatedness in the fungal population used for GWAS analysis. (a) Principal component analysis using full SNP data. PC1, PC2 and PC3 explained 1.30%, 1.12% an 0.69% of the phenotypic variation, respectively. (b) Heatmap of the kinship matrix using VanRaden method [1].

References

[1] VanRaden P.M. Efficient methods to compute genomic predictions. J Dairy Sci. 2008; 91: 4414-4423. https://doi.org/10.3168/jds.2007-0980 PMID: 18946147
