# Supplementary material for: A secreted protease-like protein in Zymoseptoria tritici is responsible for avirulence on Stb9 resistance gene in wheat
Source: PLoS Pathog. 2023 May 12;19(5):e1011376. doi: 10.1371/journal.ppat.1011376 (PMC10208482; doi:10.1371/journal.ppat.1011376)
Supplement: S3 Fig — The horizontal line indicates the genome-wide significance threshold (Bonferroni correction at α<0.05). (PDF) [file ppat.1011376.s010.pdf]

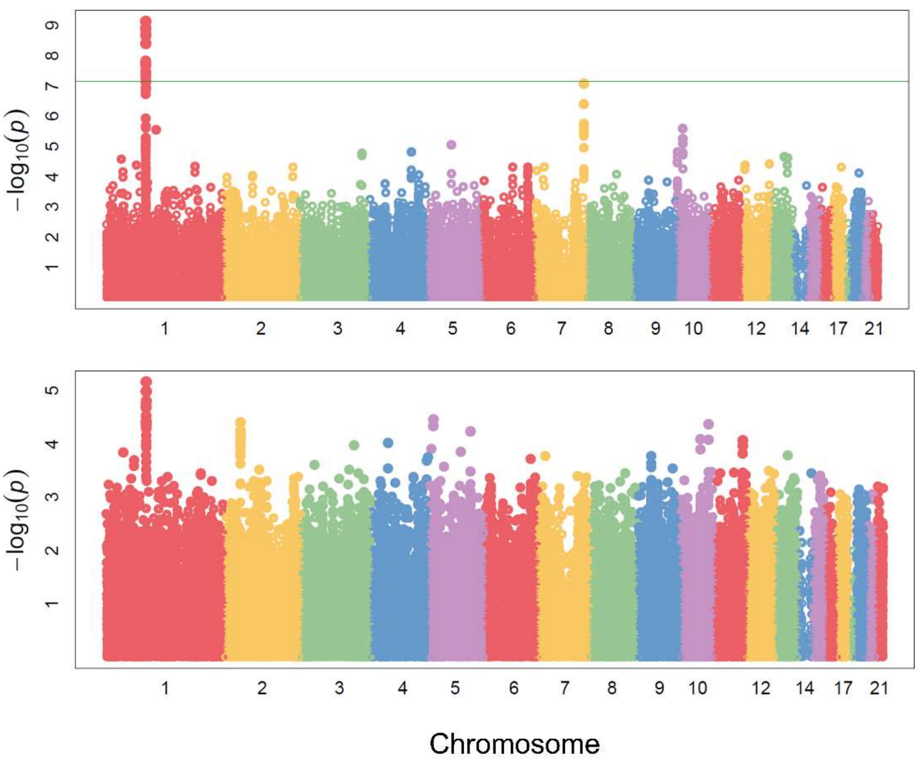

S3 Fig. Manhattan plots of PLACN (top) and PLACP (bottom) on the wheat cultivar ‘Soissons’. The horizontal line indicates the genome-wide significance threshold (Bonferroni correction at α<0.05).
