# Supplementary material for: A secreted protease-like protein in Zymoseptoria tritici is responsible for avirulence on Stb9 resistance gene in wheat
Source: PLoS Pathog. 2023 May 12;19(5):e1011376. doi: 10.1371/journal.ppat.1011376 (PMC10208482; doi:10.1371/journal.ppat.1011376)
Supplement: S4 Fig — (PDF) [file ppat.1011376.s011.pdf]

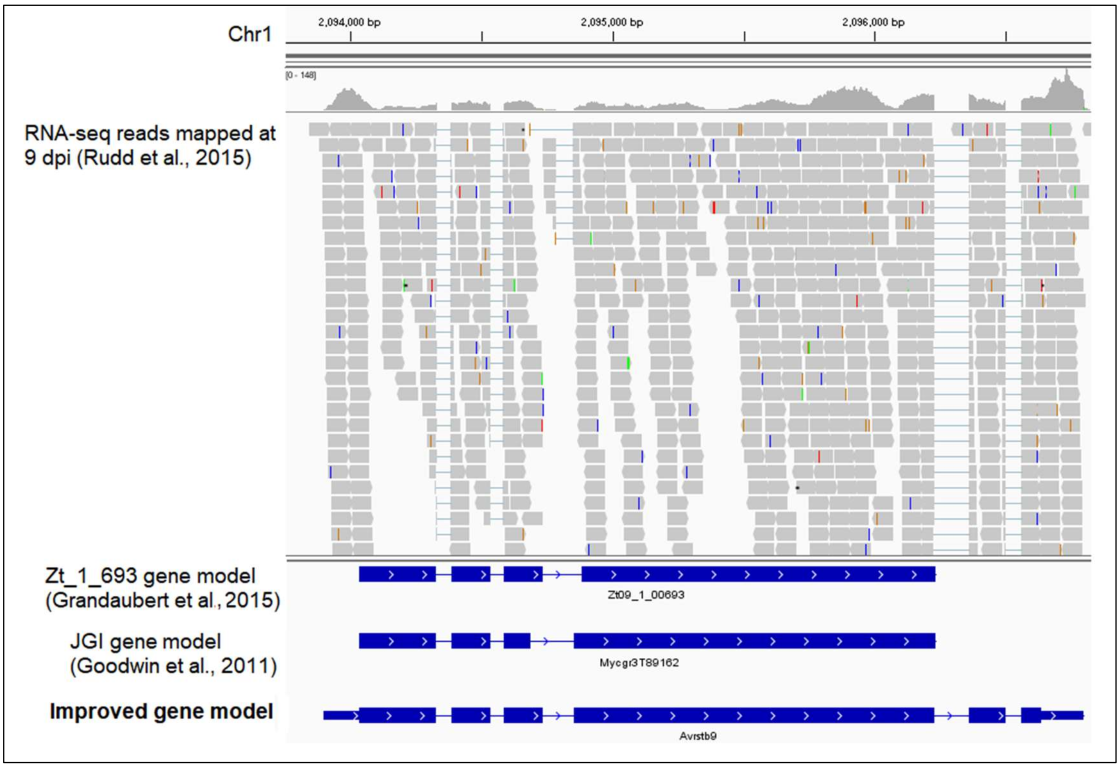

S4 Fig. Improved *AvrStb9* gene model using RNA-seq reads at 9 dpi mapped to the reference genome IPO323 [1,2].

References

[1] Goodwin SB, Ben M'Barek S, Dhillon B, Wittenberg AHJ, Crane CF, Hane JK, *et al.* Finished genome of the fungal wheat pathogen *Mycosphaerella graminicola* reveals dispensome structure, chromosome plasticity, and stealth pathogenesis. PLOS Genetics**.** 2011; 7: e1002070. https://doi.org/10.1371/journal.pgen.1002070 PMID: 21695235

[2] Grandaubert J, Bhattacharyya A, Stukenbrock EH. RNA-seq-based gene annotation and comparative genomics of four fungal grass pathogens in the genus Zymoseptoria identify novel orphan genes and species-specific invasions of transposable elements. G3-Genes Genom Genet. 2015; 5: 1323–1333. https://doi.org/10.1534/g3.115.017731 PMID: 25917918
