# Supplementary material for: A secreted protease-like protein in Zymoseptoria tritici is responsible for avirulence on Stb9 resistance gene in wheat
Source: PLoS Pathog. 2023 May 12;19(5):e1011376. doi: 10.1371/journal.ppat.1011376 (PMC10208482; doi:10.1371/journal.ppat.1011376)
Supplement: S5 Fig — (PDF) [file ppat.1011376.s012.pdf]

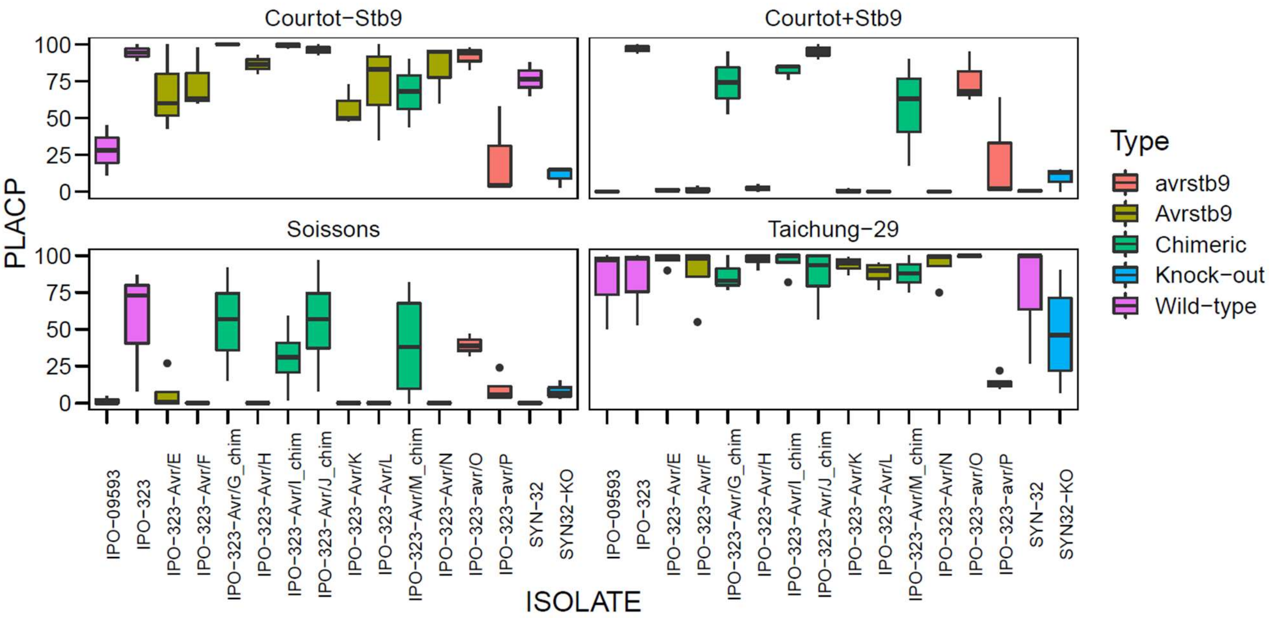

S5 Fig. PLACP of wild-type, mutant, chimeric strains and the single knock-out mutant phenotyped on cultivars carrying or not the resistance gene *Stb9* and the susceptible cultivar ‘Taichung-29’.
