# Supplementary material for: A secreted protease-like protein in Zymoseptoria tritici is responsible for avirulence on Stb9 resistance gene in wheat
Source: PLoS Pathog. 2023 May 12;19(5):e1011376. doi: 10.1371/journal.ppat.1011376 (PMC10208482; doi:10.1371/journal.ppat.1011376)
Supplement: S6 Fig — (PDF) [file ppat.1011376.s013.pdf]

(a) **(b)**

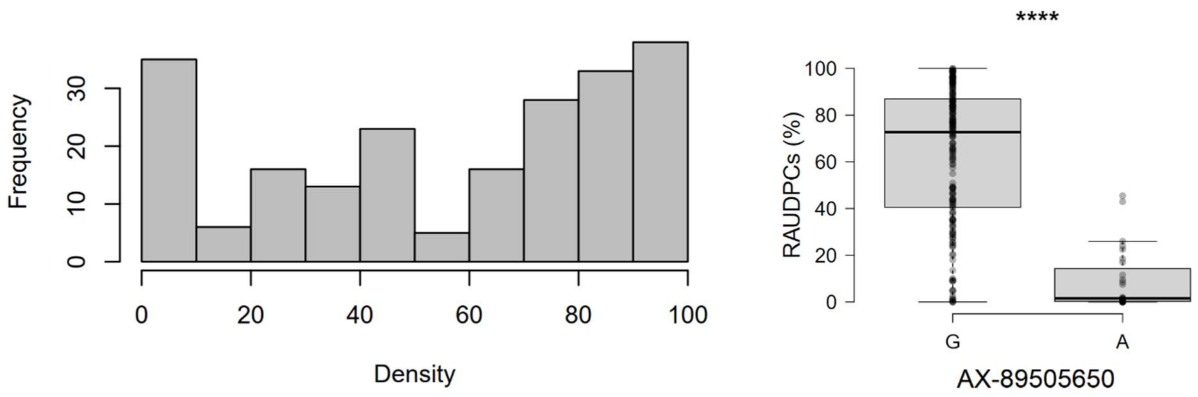

S6 Fig. Distribution of the relative AUDPC sporulating leaf (RAUDPCs) area in the panel of bread wheat cultivars (a) and the lead SNP of *Stb9* locus mapped using GWAS in the same panel (b).
