# Supplementary material for: A secreted protease-like protein in Zymoseptoria tritici is responsible for avirulence on Stb9 resistance gene in wheat
Source: PLoS Pathog. 2023 May 12;19(5):e1011376. doi: 10.1371/journal.ppat.1011376 (PMC10208482; doi:10.1371/journal.ppat.1011376)
Supplement: S7 Fig — (a) A phylogenetic tree of AvrStb9 paralogs and orthologs from Z. tritici and its relative species (Zt = Z.tritici; Zpse = Z. pseudotritici; Zbre = Z.brevis; Zard = Z. ardabilae). (b) Gene expression profile of AvrStb9 paralogs using IPO323 RNAseq data [23]. (PDF) [file ppat.1011376.s014.pdf]

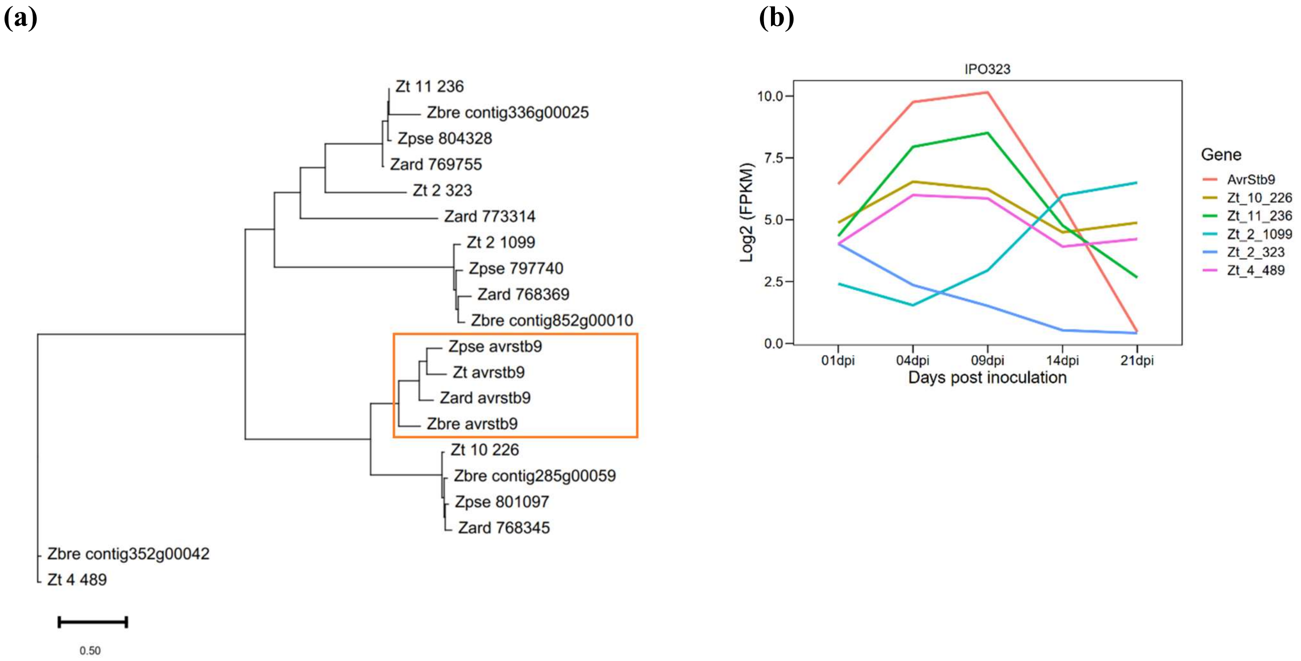

S7 Fig. *AvrStb9* homologs and their expression profile. (a) A phylogenetic tree of *AvrStb9* paralogs and orthologs from *Z. tritici* and its relative species (*Zt=Z.tritici; Zpse=Z. pseudotritici; Zbre=Z.brevis; Zard= Z. ardabilae*). (b) Gene expression profile of *AvrStb9* paralogs using IPO323 RNAseq data [1].

References

[1] Rudd JJ, Kanyuka K, Hassani-Pak K, Derbyshire M, Andongabo A, Devonshire J, *et al.* Transcriptome and metabolite profiling of the infection cycle of *Zymoseptoria tritici* on wheat reveals a biphasic interaction with plant immunity involving differential pathogen chromosomal contributions and a variation on the hemibiobiotrophic lifestyle definition. Plant Physiol. 2015; 167: 1158–1185. https://doi.org/10.1104/pp.114.255927 PMID: 25596183
