# Supplementary material for: A secreted protease-like protein in Zymoseptoria tritici is responsible for avirulence on Stb9 resistance gene in wheat
Source: PLoS Pathog. 2023 May 12;19(5):e1011376. doi: 10.1371/journal.ppat.1011376 (PMC10208482; doi:10.1371/journal.ppat.1011376)
Supplement: S8 Fig — (PDF) [file ppat.1011376.s015.pdf]

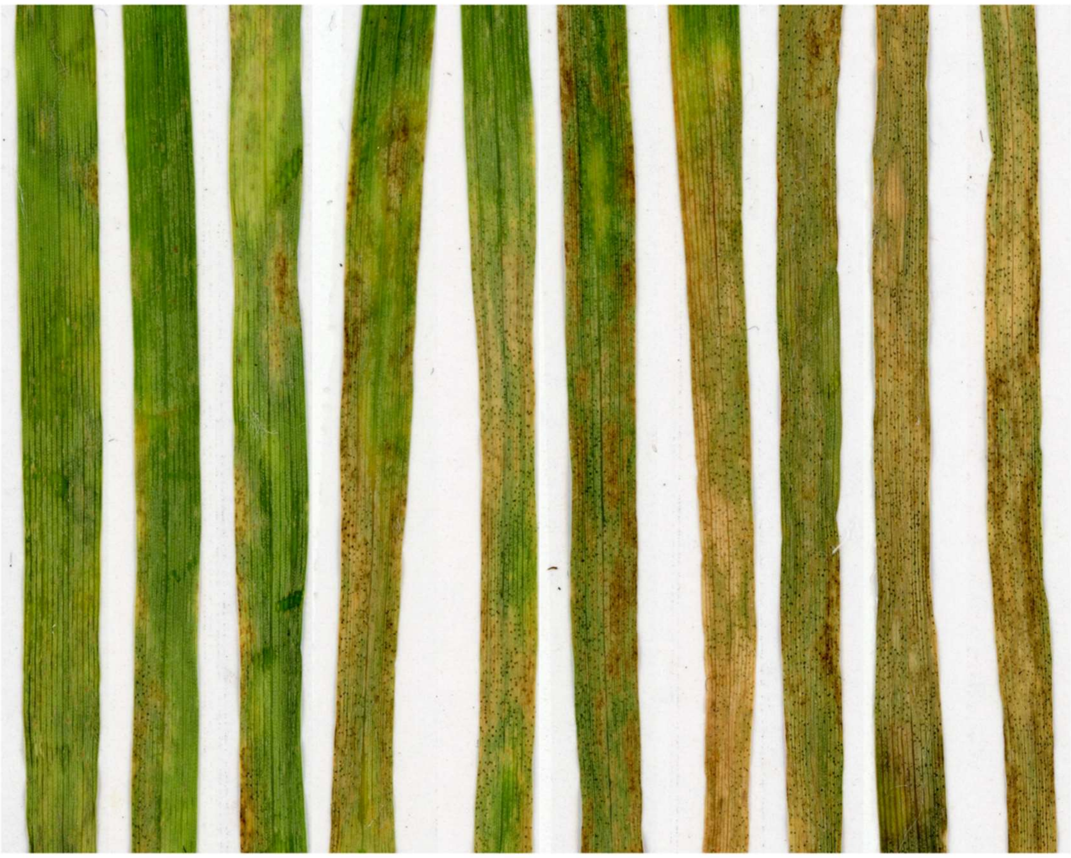

PLACN	0%	10%	20%	40%	50%	80%	90%	100%	100%	100%
PLACP	0%	5%	5%	35%	50%	65%	70%	80%	85%	100%

S8 Fig. Disease scale used for the visual assessment of percent of leaf area covered by necrosis (PLACN) and by pycnidia (PLACP) after inoculation of wheat leaves with the phytopathogenic fungus *Zymoseptoria tritici*.
