# Supplementary material for: A secreted protease-like protein in Zymoseptoria tritici is responsible for avirulence on Stb9 resistance gene in wheat
Source: PLoS Pathog. 2023 May 12;19(5):e1011376. doi: 10.1371/journal.ppat.1011376 (PMC10208482; doi:10.1371/journal.ppat.1011376)
Supplement: Attachment — Submitted filename: Amezrou et al_MS_AvrStb9_rebuttal letter.pdf [file ppat.1011376.s016.pdf]

Dear Editors and Reviewers,

On behalf of all co-authors, I would like to thank you for your comments and suggestions that greatly helped us to improve our manuscript.

We considered all points raised by the three Reviewers. You will find our answers point by point, in the letter below.

Yours sincerely,

Thierry Marcel

Part I – Summary

Please use this section to discuss strengths/weaknesses of study, novelty/significance, general execution and scholarship.

Reviewer #1:

This manuscript describes the identification of *AvrStb9* from *Z. tritici* by GWAS. For this the authors re-sequenced 103 isolates and the data were made available on NCBI. The GWAS approach lead to the identification of a specific location, but the genetic confirmation of the secreted protein to be *AvrStb9* appeared hindered by the inability of generating *AvrStb9* knock out lines suggesting that knock out of this conserved gene may be lethal. The dominant effect of an avirulence effector over virulent variants however allowed confirmation of the gene-for-gene relationship underling STB9 resistance mediated by the here identified *AvrStb9*. Although there is little functional data provided, I consider this work as well suited for Plos Pathogens, in particular with respect to the few *Avr* effectors (or effectors in general) characterized for this wheat pathogen. I have some minor comments that should improve accessibility of the data.

Reviewer #2:

The manuscript by x et al., describes the genetic mapping, population level polymorphism, expression and subsequent validation of a novel fungal avirulence gene from the globally important wheat fungal pathogen *Zymoseptoria tritici*. The authors used quantitative disease assays, whole genome sequences and corroborating *in planta* gene expression data to identify the specific polymorphisms in a putative S41 secreted protease as being responsible for recognition (or lack of) by the STB disease resistance QTL / gene *Stb9*. This *R* gene has yet to be cloned although this study also improved the resolution of its mapping and conclusively demonstrated the gene-for-gene interaction. The quality of the mapping and candidate gene selection approach is excellent, and by performing allele swaps the authors demonstrated that the protease was indeed *AvrStb9*. Unfortunately, efforts to generate knock-out mutants provided evidence for *AvrStb9* being a potential essential gene (in itself interesting), but this precluded its formal testing for a role in virulence on susceptible cultivars. However, wide population level analysis suggested that the protein is always present and functional whilst being under positive selection. Overall, this is strong work and the manuscript is very nicely written and presented.

My only concern is whether there are sufficient mechanistic insights presented. It is a pity that further characterisation of the putative avr protease was not performed. The authors themselves raise many interesting testable further questions. In particular whether protease activity was required for avr function? Or whether a small region (peptide) might confer the recognition? Is it truly an essential gene? On the other hand, if the effector had been a small

cysteine rich protein of unknown function, these questions would not be asked. On that basis I can understand the authors wish to quickly release this interesting information on a novel Avr and save detailed functional characterisation until a later date. I must therefore leave it to editorial judgement on whether the manuscript provides enough mechanistic information in its current form. I can say that I believe the novel avr identity alone will draw many citations.

Reviewer #3:

The authors describe the identification of a secreted protease from *Zymoseptoria tritici* that is likely *AvrStb9*, the protein responsible for avirulence on wheat varieties containing the *Stb9* resistance gene. This work is important as this is only the third described Avr gene from this widespread crop pathogen. The authors use a combination of techniques including genetic mapping and reverse genetics to show the likely identity of *AvrStb9*, and also the corresponding *Stb9* gene in wheat. Further, they make use of diversity present at the *AvrStb9* locus in the pathogen population to make predictions on specific residues in *AvrStb9* that are important for virulence.

Overall I found this paper to be interesting, comprehensive and well-presented. There is a good level of detail used when describing how experiments were performed and I believe these experiments to have been performed with a high level of competency. The subject matter is of great value both to the *Zymoseptoria* research community and the wider plant-microbe community given that this class of protease effector appear conserved across many species. I have some specific comments detailed below related to the presentation of some data that could help improve the manuscript:

(1) For Figure 2, it would be helpful to have leaf images showing typical symptoms of Soissons and the Courtot NILs with the different allele swap strains. It would also be helpful to see PLACN data either as part of this figure or as supplemental figure in addition to the PLACP data that is already presented. This is important as GWAS in Fig.1 found the *AvrStb9* candidate locus significance based on PLACN but not PLACP. There needs to be some additional explanation to why the GWAS found the locus based on PLACN, but the authors have used PLACP as the metric for scoring disease symptoms in subsequent experiments.

(2) *AvrStb9* structure – The section on structural biology is somewhat superficial at present. It would be helpful to show a structural prediction of *AvrStb9* protease (AlphaFold, EBI), with comparison or overlay against the S41 protease class (if experimentally validated structures are available). Highlighting of key residues for escaping *Stb9* recognition could be highlighted on this model. This would illustrate whether these key residues are separate from putative protease function. I would assume they are separate given the difficulty in obtaining a gene KO of this protease gene (as authors state). If mutations are found in protease domain, this needs to be discussed - does this not impact protease function at all, or could it impact substrate specificity. A model could also highlight active site residues, a brief literature search suggests this is a catalytic tetrad that has been partly characterized, so this should be easy to illustrate.

RESPONSE:

Unfortunately, we do not have pictures of leaves from Soissons or Courtot-NILs inoculated with virulent and avirulent strains that we could add as a Figure in our manuscript (and we do not have the possibility to produce such pictures at the moment). However, we added a Supplementary Figure 8 with typical symptoms of the wheat-*Z. tritici* interaction representing the scale that was used to score the disease.

The GWAS found the AvrStb9 locus with both PLACN and PLACP, however PLACP failed to reach the genome wide significance; this is explained lines 142-146 in the manuscript. We now added PLACN data to Figure 2 so the reader will be able to visualize the effect of the interaction on both disease traits.

We have now run AlphaFold and obtained a structural prediction of AvrStb9 (avirulent) and avrStb9 (virulent) proteins (see new Figure 5). Please, read below our complete answer on this topic to Reviewer #2.

Part II – Major Issues: Key Experiments Required for Acceptance

Please use this section to detail the key new experiments or modifications of existing experiments that should be absolutely required to validate study conclusions.

Generally, there should be no more than 3 such required experiments or major modifications for a "Major Revision" recommendation. If more than 3 experiments are necessary to validate the study conclusions, then you are encouraged to recommend "Reject".

Reviewer #1:

One thing I consider as major issue in the current version of the manuscript is the insistence of the authors (abstract, introduction and discussion) that AVR effectors are generally considered small secreted proteins without functional domains. This is a very outdated and narrow view and should as such not be present in a publication with relatively broad readership.

Example: Line 40 cont: It is commonly assumed that most Avr genes encode small secreted proteins, with no predicted functional domain and no sequence homology in other species. But the use of methods without a priori on the function or structure of genetic determinants may allow to identify Avr genes coding for proteins not falling into this category.

- I assume the authors try to differentiate their here generated data (*AvrStb9* is rather large and carries predicted protease domains) from previous reports by using the statement as selling point. However, this is simply not correct. First, only fungal effectors are frequently referred to as small secreted proteins, but even for fungi, not all characterized effectors are small. Hundreds of Avr genes have been predicted to carry functional domains. The best studies ones in this sense are likely bacterial Avrs, but also fungal Avrs were shown to have functional enzymatic activity (eg. *Z. tritici AvrStb6*). Similarly, the discussion associated does not give any deep insight and is rather a selective list of examples.

RESPONSE: We fully agree with this statement and we are thankful for pointing this out. We have now revised all parts of the manuscript (summary, introduction and discussion) where this was stated, and provide particularities of the new *Avr* gene more explicitly. For example, lines 76-88 in the Introduction

- Considering the limited knowledge on functional studies associated with (avirulence) effectors provided here, I suggest to not go further into this topic or to comment on effector functions/domain functions. It will be sufficient to mention that unlike many other effectors, this one here appears large in size. (Many effectors actually appear to have predicted functional domains, especially when looking at their predicted structures. However, as for here-identified *AvrStb9*, it was not (yet) clarified weather these domains are functional. (This is generally often ignored). For this reason, I also suggest to remove the reference to 'protease-like' from the title.)

RESPONSE: We agree with Reviewer #1 that the function of AvrStb9, particularly its protease activity, has not yet been verified experimentally. However, we believe that it remains important to mention the domains and possible associated functions that we identified through homology searches at the sequence and protein structure levels. We revised our manuscript being extremely cautious in suggesting these findings.

Reviewer #2: (No Response)

Reviewer #3: (No Response)

Part III – Minor Issues: Editorial and Data Presentation Modifications
Please use this section for editorial suggestions as well as relatively minor modifications of existing data that would enhance clarity.

Reviewer #1:

Clarity: For generating easy data access to the readership, I suggest the authors to indicate in the graphs (Fig 2) if an isolate is known to be avirulent or virulent on *Stb9* (eg….. IPO323 (Stb6_vir) ….).

RESPONSE: As suggested, we have added "Virulent" and "Avirulent" suffixes to the isolates in Fig. 2.

Fig. 2 a IPO09593 appears generally weakly virulent also in -stb9 lines. With the statistics applied here (I assume it is an anova test) the data suggests no statistical difference between the virulence of the isolate between +Stb9 and -Stb9 lines. This may confuse the readership but is likely simply an artefact because of the way the stats are applied. A non-parametric test (for example Kruskal-Wallis followed by Dunn's test) is more appropriate here (either way, the test applied should be indicated in the legend). It may also be useful to separately analyses the differences for each wheat background (if so, please also indicate in the legend).

RESPONSE: We totally agree that the way this data and statistical significance are presented may confuse the readership. We choose to apply the post-hoc Tukey's HSD test for mean comparisons between isolate groups because it accounts for multiple comparisons and is not inflated by Type I errors. It was done separately for each wheat background. To avoid the confusion, we have indicated the details of the applied statistics explicitly in the figure legend, and improved the figure by separating the plots for each wheat background.

Clarity: Define PLACP in figure legends and maybe indicate y axis as 'necrosis (PLACP)'.

RESPONSE: We have now defined PLACP in figure legends and added 'Sporulation (PLACP)' or 'Necrosis (PLACN)' in the y axis of corresponding graphs.

Line 51: please change to 'for SOME OF the most….'.

RESPONSE: We have corrected this sentence and added the suggested word.

Line 53: play critical roleS.

RESPONSE: We have now corrected the sentence.

Line 107: are avirulent on 'soissons'.

RESPONSE: We have rephrased this sentence.

Discussion: The Avr gene is expressed at the timepoint of lifestyle transitioning from bio to necrotrophy. At this point of our knowledge, this is rather rare. Only few Avrs have been characterized for hemi-biotrophs, but the best studies ones (eg. from M. oryzae) are recognized during the biotrophic phase. This cuts off infection early on. And in agreement these effectors induce R-gene dependent cell death in heterologous overexpression assays. The late recognition of AvrStb9 could suggest an host STB9 resistance function that inhibits transition to necrotrophy, i.e. inhibition of cell death. This is purely speculative at this stage as heterologous assays are not shown (and I am not asking the authors to perform these for this manuscript), but it may be an interesting point to discuss in the manuscript so that assays testing such hypothesis may be considered in the future. This point could replace the statements about effectors generally being SSP.

RESPONSE: We fully agree with this suggestion and we have added few sentences in the discussion addressing this specific point, lines 326-329.

Reviewer #2:

-It is stated several times that recognition triggers "a strong immune response" but no responses are actually measured, only lack of disease symptoms. How do you know that a low

or moderate immune response might not already be sufficient to prevent the disease? Suggest rephrase.

RESPONSE: This is an interesting remark. We have now rephrased when necessary and removed the word "strong" whenever it appeared.

-Whilst detailed functional analysis of the putative protease is planned for the future it would have been nice to view the polymorphic residues in the context of the positions of essential amino acids likely required for catalytic activity (eg using NCBI conserved domain databases or similar) ie it would be nice the see the position of polymorphic amino acids in greater resolution than the current Fig 3C. Similarly, it would be nice to run alphafold, or a structural prediction software, to see if the polymorphism likely affects folding or overall protein structure or alters accessibility to the catalytic domain.

RESPONSE: Thank you for these valuable suggestions. We have now run AlphaFold and obtained a structural prediction of AvrStb9 (avirulent) and avrStb9 (virulent) proteins (see new Figure 5). We could search for structural homologs in the full Protein Data Bank (PDB), overlay the predicted structures of the virulent and avirulent forms and compare the localization of the five residues under selection between both forms. It allowed us to confirm that the secreted protease CPAF is the closest structural homolog to AvrStb9 and to pinpoint the potential importance of residue S525 in escaping recognition by Stb9. As our colleague Clémentine Louet helped us to realize these new analyses, we propose to add her as a co-author to our manuscript.

-Given that the journal has a broad readership (including many studying disease of animals etc) I think it would be useful to include at least a few photos of infected leaf tissues so that a non-specialist reader can make sense of the quantitative data such as that shown in Fig2. Maybe in supplementary if not in main? Within the Zymoseptoria community we are aware of how these things look, but it is certainly not true for researchers from disciplines further afield.

RESPONSE: Thank you for this suggestion, also made by Reviewer #3. We are not able to provide leaf images with symptoms of Soissons and the Courtot NILs with the different allele swap strains because we do not have them. Moreover, as our laboratory has recently moved to new facilities, we do not have the possibility to repeat the inoculations and take pictures at the moment. However, we are now providing as Supplementary Figure 8, pictures of symptomatic wheat leaves representative of the disease scale that we used to evaluate the interactions.

-Line 345 discussion highlights some potential means by which *avrStb9* and *Stb9* might interact but does not consider the "Guard" model or that the protease might produce a peptide which is recognised by *Stb9*???

RESPONSE: This was certainly an oversight and we are thankful for pointing this out. We have now included the "guard" model and the peptide-recognition hypothesis in the discussion, as a potential means by which the *AvrStb9* and *Stb9* might interact, lines 403-406.

-The authors are correct in stating that large enzymes are not typical for fungal avr's but they are quite well known to be recognised by receptor-like proteins during PTI (for examples see Plant Cell. (2004) 16: 1604–1615; EMBO J (2002) 21(24):6681-8). Indeed, there is now a well-established blurring between ETI and PTI (Plant Cell (2011) 23(1):4-15). PAMP's are also often essential and cannot be lost or mutated (alike *AvrStb9*???). It would be nice to perhaps have a little more discussion on the possibility that *Zymoseptoria* might co-opt more typical PTI mechanisms to establish ETI?

RESPONSE: The discussion on the role played by AvrStb9 in PTI or in ETI, and the blurred line between both types of immunity, is a very interesting one. PAMPs are usually described as being evolutionarily conserved structures which are functionally important. It seems that AvrStb9 cannot be lost, as suggested by our difficulty to obtain KO mutant. However, the sequence of AvrStb9 has undergone diversification, and all mutations observed (including an 18 bp deletion) are not necessary to escape recognition by Stb9. In that sense, AvrStb9 doesn't

resemble what is expected to be a PAMP. Nevertheless, we are not feeling comfortable to add this discussion in our manuscript for the moment, as we simply do not know enough on the molecular functions of AvrStb9 neither on the corresponding resistance gene *Stb9*. We hope that our work will help to answer these questions and to address this topic in a near future.

Reviewer #3:

I found several presentational errors related to figures and their associated legends;

Label for Figure 1B, D is out of place on figure. Legend confuses parts B and C.

Figure 2 has no part B, labelled only as parts A, C and D.

Figure 3 sections B and C don't match with legend.

RESPONSE: Thank you for pinpointing these errors in the labelling of figures. We have now corrected them.
